# The Carcinogenic Potential of Bisphenol A in the Liver Based on Transcriptomic Studies

**DOI:** 10.3390/cancers15205014

**Published:** 2023-10-17

**Authors:** Marta Wiszpolska, Ewa Lepiarczyk, Mateusz A. Maździarz, Łukasz Paukszto, Karol G. Makowczenko, Aleksandra Lipka, Elżbieta Łopieńska-Biernat, Krystyna Makowska, Sławomir Gonkowski, Paulo Correia-de-Sá, Marta Majewska

**Affiliations:** 1 Department of Human Physiology and Pathophysiology, School of Medicine, University of Warmia and Mazury in Olsztyn, 10-082 Olsztyn, Poland; marta.majewska@uwm.edu.pl; 2 Department of Botany and Nature Protection, Faculty of Biology and Biotechnology, University of Warmia and Mazury in Olsztyn, 10-727 Olsztyn, Poland; mazdziarzm@gmail.com (M.A.M.); pauk24@gmail.com (Ł.P.); 3 Department of Reproductive Immunology and Pathology, Institute of Animal Reproduction and Food Research of PAS, 10-748 Olsztyn, Poland; k.makowczenko@pan.olsztyn.pl; 4 Institute of Oral Biology, Faculty of Dentistry, University of Oslo, 0372 Oslo, Norway; aleksandra.lipka@odont.uio.no; 5 Department of Biochemistry, Faculty of Biology and Biotechnology, University of Warmia and Mazury in Olsztyn, 10-719 Olsztyn, Poland; ela.lopienska@uwm.edu.pl; 6 Department of Clinical Diagnostics, Faculty of Veterinary Medicine, University of Warmia and Mazury in Olsztyn, 10-957 Olsztyn, Poland; krystyna.makowska@uwm.edu.pl; 7 Department of Clinical Physiology, Faculty of Veterinary Medicine, University of Warmia and Mazury in Olsztyn, 10-957 Olsztyn, Poland; slawomir.gonkowski@uwm.edu.pl; 8 Laboratório de Farmacologia e Neurobiologia, Center for Drug Discovery and Innovative Medicines (MedInUP), Instituto de Ciências Biomédicas de Abel Salazar, Universidade do Porto, 4050-313 Porto, Portugal; farmacol@icbas.up.pt

**Keywords:** bisphenol A, liver, mitochondrial dysfunction, hepatocellular carcinoma, RNA-Seq

## Abstract

**Simple Summary:**

Bisphenol A (BPA), an endocrine-disrupting chemical extensively used in the production of everyday products, profoundly affects human homeostasis and well-being. Given the liver’s critical role in toxins’ first passage detoxification, it may become highly susceptible to BPA harmful effects. The present study aimed at investigating changes in the liver transcriptomics caused by oral exposure to BPA in mice. Our findings may contribute to clarifying the impact of BPA on gene expression in mice livers to predict the molecular mechanisms underlying BPA-related hepatic toxicity and carcinogenic effect.

**Abstract:**

Bisphenol A (BPA) is an environmental toxin widely used in the production of polycarbonate plastics. A correlation exists between BPA tissue contamination and the occurrence of pathological conditions, including cancer. First-passage detoxification of high BPA amounts in the liver promotes hepatotoxicity and morphological alterations of this organ, but there is a lack of knowledge about the molecular mechanisms underlying these phenomena. This prompted us to investigate changes in the liver transcriptomics of 3-month-old female mice exposed to BPA (50 mg/kg) in drinking water for 3 months. Five female mice served as controls. The animals were euthanized, the livers were collected, and RNA was extracted to perform RNA-seq analysis. The multistep transcriptomic bioinformatics revealed 120 differentially expressed genes (DEGs) in the BPA-exposed samples. Gene Ontology (GO) annotations indicated that DEGs have been assigned to many biological processes, including “macromolecule modification” and “protein metabolic process”. Several of the revealed DEGs have been linked to the pathogenesis of severe metabolic liver disorders and malignant tumors, in particular hepatocellular carcinoma. Data from this study suggest that BPA has a significant impact on gene expression in the liver, which is predictive of the carcinogenic potential of this compound in this organ.

## 1. Introduction

Bisphenol A (BPA) is a substance commonly found in our environment. This ubiquitous chemical was first synthesized in 1891 by Alexander P. Dainin through the condensation of two phenol molecules and one molecule of acetone, in the presence of a catalyst—hydrogen chloride or ion-exchange resin [1,2]. The available data suggest that approximately 8 million tons of BPA are produced worldwide each year [3]. Due to its very good mechanical properties, the low adsorption of moisture, and thermal stability, BPA is a constituent of synthetic polymers that are applied in the production of food containers, bottles, toys, dentistry products, laminated flooring, paints, and electronic devices [1,4]. Additionally, it is utilized in the manufacturing process of thermal paper used in shop receipts, tickets, envelopes, and even books [1,5]. Nevertheless, it is important to note that starting from 2 January 2020, the European Chemicals Agency (ECHA) imposed restrictions on the sale of thermal paper containing a concentration of BPA exceeding 0.02% by weight [6].

The human body may be contaminated with BPA by lifelong exposure to this xenoestrogen endocrine disruptor through oral intake (food from plastic and metal packaging, dental materials), dermal absorption (shop tickets, envelopes, books), and/or inhalation (dust from laminate flooring, paints) [7,8]. The issue of BPA food contamination is particularly worrisome as this chemical is released from container polymers into meals or water when products are heated or exposed to ultraviolet light [9].

Due to its lipophilic characteristics, BPA is rapidly absorbed and can accumulate in multiple organs of the human body, such as kidneys, liver, adipose tissue, and placenta [1]. While the half-life of BPA is roughly 4.5 days in water and less than one day in the air due to its low volatility, the potential risk of its accumulation in the human body arises from prolonged and repeated exposure over extended periods [10]. After enteric absorption, BPA is mostly metabolized in the liver in a process initiated in the endoplasmic reticulum, resulting in the formation of glucuronide and sulfate conjugates [11,12]. The dominant metabolite of BPA, known as BPA glucuronide conjugate, is primarily eliminated through the bile. This fact, coupled with the chemical stability of the conjugate, suggests its potential as a valid biomarker for assessing exposure [13,14]. 

Considering that the liver is a critical player in the detoxification and metabolism of many chemical substances, concerns arise about the disruption of the normal functioning of this organ upon lifelong exposure to BPA [3]. BPA metabolites contribute to the formation of DNA adducts and cause the downregulation of antioxidant genes, thus, promoting hepatotoxicity by allowing reactive oxygen species (ROS) accumulation [15,16,17]. Transcriptomic studies indicate that BPA may influence the metabolism of lipids through the overexpression of lipoprotein lipase, β-acetyl-coenzyme A carboxylase, and fatty acid synthase [18,19]. Additionally, BPA exposure has been associated with hepatocellular carcinoma (HCC) [20] and non-alcoholic fatty liver disease (NAFLD) [21].

Despite data available in the literature, there is a lack of information regarding the molecular mechanisms underlying BPA-induced hepatotoxicity. This prompted us to investigate the liver transcriptomic signature by targeted bioinformatic analysis in living mice exposed to oral BPA for three months.

## 2. Materials and Methods

### 2.1. Laboratory Animals

The study was performed on ten 3-month-old female mice (Mus musculus, C67BL6/J/CMDB strain) with an average body weight of 30 g. They were maintained under a controlled temperature of 22 ± 20 °C, humidity 55 ± 10%, and 12:12 h light–dark cycle. Mice were kept in the animal house (at the Faculty of Veterinary Medicine, University of Warmia and Mazury in Olsztyn, Poland) with free access to food and water. The experiment was performed following the guidelines of the local ethics committee for animal experimentation in Olsztyn, Poland (affiliated with the National Ethics Committee for Animal Experimentation, Polish Ministry of Science and Higher Education; decision No. 46/2019). The animals were randomly divided into two groups. Mice were given the same food and were weighed weekly. During the experiment, five mice served as controls (CTR), and these animals did not undergo any experimental procedures. BPA in the drinking water at a dose of 50 mg/kg b.w. for 3 months was given to the other 5 mice and they served as an experimental group (BPA-treated). This dose is considered a lowest observed adverse effect level (LOAEL) for this species [22,23]. The BPA dose was gradually increased by weighing mice every week. The animals were decapitated after 3 months [24]. The livers were immediately removed after death and stored under liquid nitrogen at −80 °C until further experimental procedures.

### 2.2. RNA Extraction, Library Construction, and High-Throughput Transcriptome Sequencing

The total RNA was isolated from the livers of both groups using the mirVanaTM miRNA Isolation Kit with phenol according to the manufacturer’s protocol (Thermo Fisher Scientific, Waltham, MA, USA). To measure the quantity and quality of total RNA isolates, a 2100 Bioanalyzer (Agilent Technologies, Santa Clara, CA, USA) with a 6000 Nano LabChip Kit was used. For the construction of the RNA-seq libraries, samples with the highest RNA integrity number (RIN) values and concentrations were chosen. Briefly, 1 µg of total RNA was used for library construction by the Illumina TruSeq mRNA LT Sample Prep kit (Illumina, Inc., San Diego, CA, USA). Primary mRNA was purified using poly-T-attached magnetic beads, and then divalent cations were used for fragmentation of the templates. To generate the initial complementary DNA (cDNA) strands, the fragmented RNA was amplified with SuperScript II reverse transcriptase (Invitrogen, Waltham, MA, USA) and random primers. In the subsequent step, the second-strand cDNA was synthesized using DNA polymerase I and RNase H. To construct the final cDNA libraries, the PCR products were purified and enriched. Quantity of the RNA-seq libraries was assessed with real-time PCR (KAPA Library Quantification kits for Illumina Sequencing platforms). The quality of the libraries was determined using the TapeStation D1000 ScreenTape (Agilent Technologies, Waldbronn, Germany). Next, indexed libraries were sequenced on the NovaSeq 6000 platform (Illumina, San Diego, CA, USA). Library preparation and sequencing procedure were outsourced to external company (Macrogen, Geumcheon-gu, Republic of Korea).

### 2.3. In Silico Profiling of Liver Transcriptome Affected by BPA

#### 2.3.1. Processing and Mapping of Raw Reads

The raw high-throughput sequencing data obtained from the NovaSeq 6000 system underwent an assessment based on established quality control criteria using the FastQC software version 0.11.7 [25]. The paired-end reads (2 × 150 bp, with stranded orientation) were subjected to a trimming process, where Illumina adaptors were detected within the sequences and low-quality reads (PHRED cut-off score < 20) were removed from subsequent analysis using Trimmomatic software v. 0.38 [26]. The resulting 120 bp trimmed reads were aligned to the mouse reference genome, specifically, according to Genome Reference Consortium Mouse Build 39annotation (GRCm39), utilising STAR software v. 2.7.10a [27]. Alignments involving multiple instances of the same sequence were disregarded for further analysis. The incorporation of the StringTie v. 2.2.1 pipeline allowed for a re-evaluation of the Genome Reference Consortium annotations, resulting in the identification of novel sequences on regions of intergenic expression [28]. Whole transcriptome high-throughput sequencing (RNA-seq) of BPA libraries was conducted to identify the expression profiles of differentially expressed genes (DEGs), differentially expressed long non-coding RNA (DELs), and differential alternative splicing events (DASs).

#### 2.3.2. Detection of Differentially Expressed Genes and Long Non-Coding RNAs and Interaction Analyses

The analysis focused on identifying differentially expressed protein-coding transcripts. These transcripts were organized based on their genomic position and tagged as transcriptionally active regions (TARs). To perform the differentially expressed analysis, the DESeq2 tool v. 1.36.0 [29] was utilized, employing a negative binomial generalized linear model. Only TARs whose expression modification patterns reached the presumed binary logarithm of fold change (log2FC) cutoff level (absolute log2FC > 1) and significance threshold (adjusted *p*-value < 0.05) were included in further analyses. TARs located within the genomic region of protein-coding genes were categorized as DEGs, while those occurring in regions of long non-coding RNAs were classified as DELs. The DEGs–DELs potential correlations were estimated by co-expression analysis and the pairs located on different chromosomes but showing similarity of transcriptional profiles were characterized as trans-interactions based on Pearson’s correlation coefficient (absolute *r* value > 0.8 and *p*-value < 0.05).

#### 2.3.3. Alternative Splicing Events and Differential Analysis

The replicate multivariate analysis of transcript splicing (rMATS v.3.2.5) was used to find differences in alternative splicing (AS) events among examined groups and the analysis was based on RNA-seq raw reads mapped to the reference *Mus musculus* genome [30]. The examined AS events included: alternative 5′ splice site (A5SS), alternative 3′ splice site (A3SS), mutually exclusive exons (MXE), retained intron (RI), and skipped exon (SE). The percent of splicing inclusion (PSI) values was calculated for all AS events according to the reads aligned to the spliced junction sites. The differential alternative splicing (DAS) events were statistically tested (FDR < 0.05) for each subject of the experimental and the control group. Moreover, DAS events were filtered according to the differential level of the ΔPSI > 0.1. To draw volcano and heatmap plots, R Bioconductor packages (http://www.R-project.org/, accessed date: 15 January 2023; v.4.1.1), i.a., ggplot2 (Wickham H. 2016, v.3.3.5), and circlize (Gu. Z. 2014, v.0.4.15) were applied.

#### 2.3.4. Functional Annotations of DEGs, DELs, and DASs 

The obtained liver gene signatures, DEGs, DELs, and DASs, were scanned according to Gene Ontology Consortium (GO) annotations [31,32] using g:Profiler software [33]. Biological processes (BP), cellular components (CC), and molecular functions (MF) were annotated as ontological terms for the essential genes. The enrichment analysis (adjusted *p*-value < 0.05) was applied to uncover ontology and pathway annotations regulated by DEGs, DELs, and DASs. To visualize the contribution of the identified DEGs, DELs, and DASs to liver function, those events were highlighted using the circlize R packages.

### 2.4. Real-Time PCR

The mRNA levels of specific transcripts were determined by real-time PCR. Primer3Plus software was used to design primers for target genes based on the sequences indicated in Table S1 [34]. The cDNA was synthesized using the Applied Biosystems™ High-Capacity cDNA Reverse Transcription Kit (Thermo Fisher Scientific, Vilnius, Lithuania) according to the manufacturer’s instructions. Real-time PCR was performed using the Applied Biosystems™ PowerUp™ SYBR™ Green Master Mix (Thermo Fisher Scientific, Vilnius, Lithuania) on the QuantStudio™ 3 Real-Time PCR System (Applied Biosystems™, Thermo Fisher Scientific Inc., Waltham, MA, USA). Four technical replicates were performed for each biological sample. The expression level of each gene was determined by the comparative Pfaffl method [32], in which gene expression in treated samples was changed by a factor compared with control samples and normalized to endogenous reference genes (UBC, GenBank NM _019639.4, and ACTB GenBank NM _007393.3) (relative quantification RQ = 1). Results were expressed as means of biological replicates ± standard deviations. Statistical analysis was performed using Prism 8 software (GraphPad Software Inc, San Diego, CA, USA) with a two-tailed Student’s t test. *p* values < 0.05 were considered statistically significant when <0.0332 (*), <0.0021 (**), <0.0002 (***), and <0.0001 (****) [35].

## 3. Results

### 3.1. Liver Transcriptomic Statistics and the Abundance of Expression Profiles

Overall, as a result of sequencing, 63.318 mln raw reads were obtained. The filtration procedure removed 9.187 mln reads with a low-quality score and the trimming procedure clipped out Illumina adaptor sequences. The surviving 49.619 mln paired-end reads were mapped to the *Mus musculus* reference genome (GRCm39). The results of the mapping process were applied to the further identification of the DEGs, DELs, and DASs. Uniquely mapped reads contained an average of 92.24% out of all processed sequences. According to the gene structure, 68.67% of paired-end reads’ nucleotides were aligned to the coding sequence (CDS), 27.00% to the untranslated regions (UTR), 2.62% to the intronic sequences, and 1.70% to the intergenic localizations. A numerical summary of the initial stages of sequencing data processing is shown in Table 1. The raw reads obtained during this study have been submitted to the European Nucleotide Archive under accession no. PRJEB65086.

### 3.2. Transcriptomic Differences in the Liver after Oral Exposure to BPA

**Figure 1:**
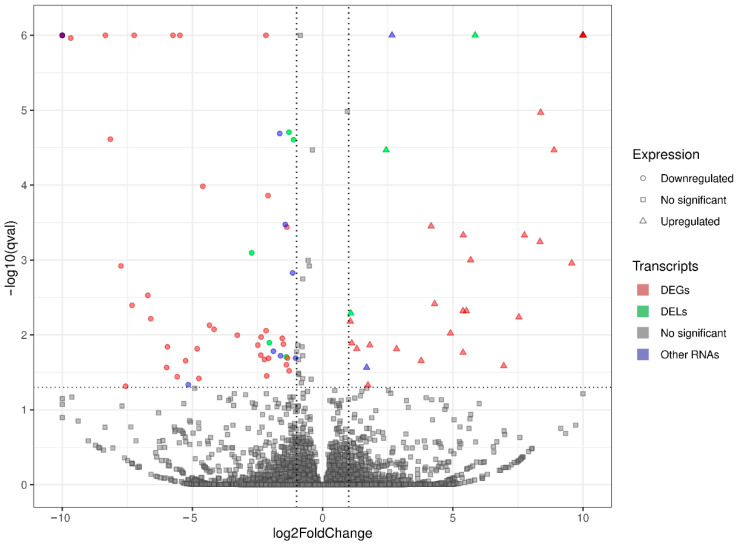
Volcano plot presenting the expression changes measured by log2 fold change (log2FC) level within gene expression profiles in BPA-treated versus control experimental comparison. The difference values of each gene are presented on the *X*-axis, and the negative logarithmic adjusted *p*-value (*q*-value) is presented on the *Y*-axis. The horizontal dotted line is equal to the negative logarithmic value of q cut-off (0.05) and two vertical lines are equal to the absolute value of log2FC (1.0). Colored points represent different types of significant differentially expressed genes (DEGs), long non-coding RNAs (DELs), and other RNAs (DE-RNAs) and gray points represent non-significant genes.

**Figure 2:**
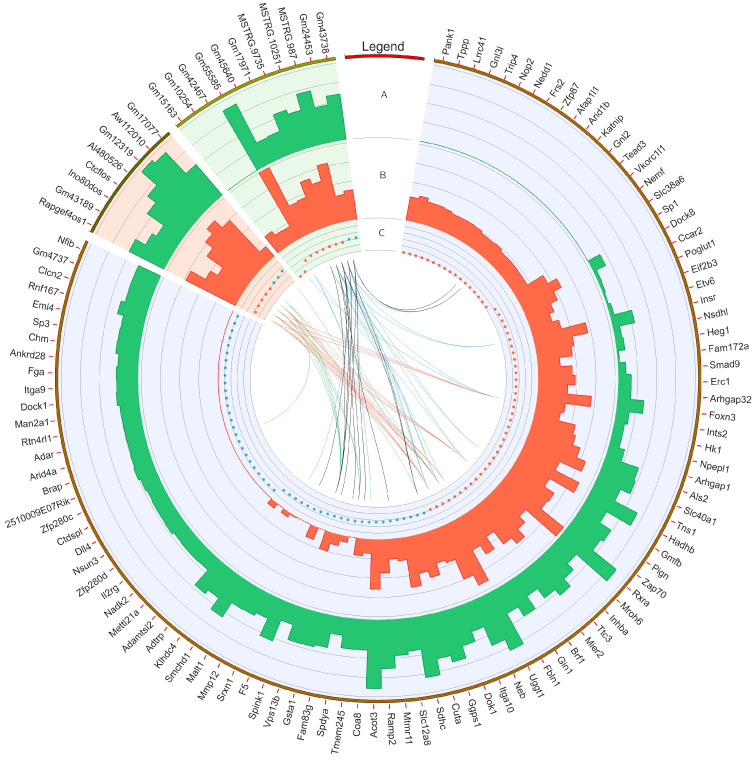
Circular heatmap visualization of differentially expressed genes (DEGs), long non-coding RNAs (DELs), and other DE-RNAs in BPA-affected and control libraries. The DEGs, DELs, and other DE-RNAs were highlighted by blue, red, and green backgrounds, respectively. The first track shows the gene ID for particular transcripts/genes. In the second (**A**) and third tracks (**B**), the bars describe the summarized counts values of BPA-treated (green bars) and CTR (red bars). The fourth track (**C**) depicts down- (red dots) and upregulated (blue dots) transcripts. The internal links merge expression profiles of DEGs and other non-coding RNAs according to the Pearson correlation coefficient (absolute *r* value > 0.8). The red and blue links join positively correlated DEGs and non-coding RNAs. The black and green lines show negative correlations.

Screening RNA-seq data for differential gene expression analysis revealed that the liver transcriptome affected by oral exposure to BPA was associated with 120 differentially expressed TARs. Among them, 101 TARs encoding protein sequences were classified as DEGs. In total, 48 DEGs were upregulated and 53 were downregulated under the influence of BPA. Estimated DEGs’ log2FC values range from −26.14 (*PANK1*, encoding the pantothenate kinase regulating the biosynthesis of coenzyme A) to 25.62 (*NFIB*, regulating the expression of transcription factors, like TGF-β1, involved in differentiation). The deep transcriptome analysis reveals eight long non-coding RNA (DELs) under BPA influence, within which five are upregulated (highest log2FC = 5.86—*GM17077*) and three downregulated (lowest log2FC = −2.72—*RAPGEF4OS1*). An overview of the expression profiles of all DEGs, DELs, and other TARs is represented as a volcano plot (Figure 1) and a heatmap supplemented with trans-interactions information (Figure 2). Statistics on identified DEGs, DELs, and other differentially expressed RNAs (DE-RNAs) are summarized in Table S2.

The co-expression analysis reveals 27 DEG−DEL trans-interactions. Identified events show the mediation of six DELs in the regulation of nine DEGs. The majority of DEG−DEL trans-actions (19) are positively correlated, although only 8 show a negative correlation of expression (involving DEGs: *DOK1*, *FBLN1*, *SPDYA,* and *SPINK1*, and DELs: *AI480526*, *CTCFLOS*, *GM17077*, *GM43189*, and *INO80DOS*). Detected trans-interactions details are summarized in Table S3.

### 3.3. Transcriptomic Alternative Splicing Signatures of the Liver after Oral Exposure to BPA

**Figure 3:**
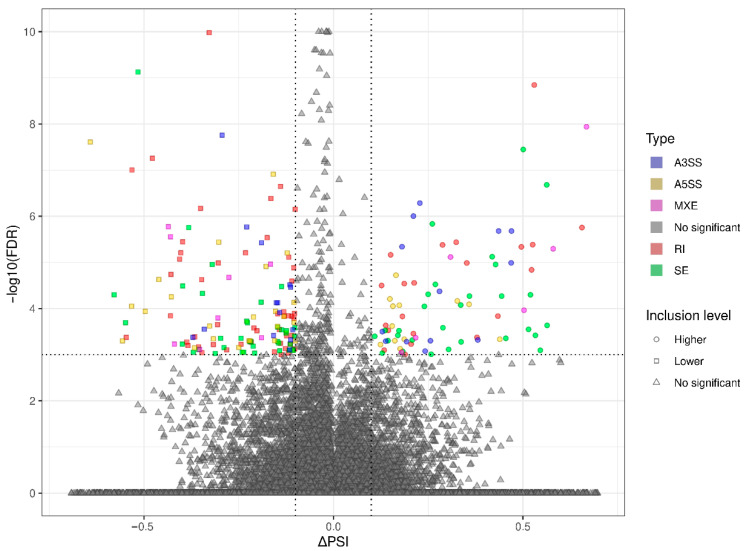
Volcano plot showing the inclusion level differences (ΔPSI; *X*-axis) against the statistical significance (−log10FDR; *Y*-axis) of differential alternative splicing events (DASs) identified within genes of murine BPA-affected liver vs. control samples. The horizontal dotted line is equal to the negative logarithmic value of the FDR cut-off (0.05) and two vertical lines are equal to the absolute value of 0.1 ∆PSI. The colors of the dots indicate specific types of DAS: alternative 3′ splice site (A3SS—blue), alternative 5′ splice site (A5SS—yellow), mutually exclusive exons (MXE—purple), retained intron (RI—brown), skipping exon (SE—green), and not a significant event (gray). Uncolored figures denote ΔPSI values (circle—higher inclusion level in BPA, square—higher inclusion level in CTR, triangle—not significant).

**Figure 4:**
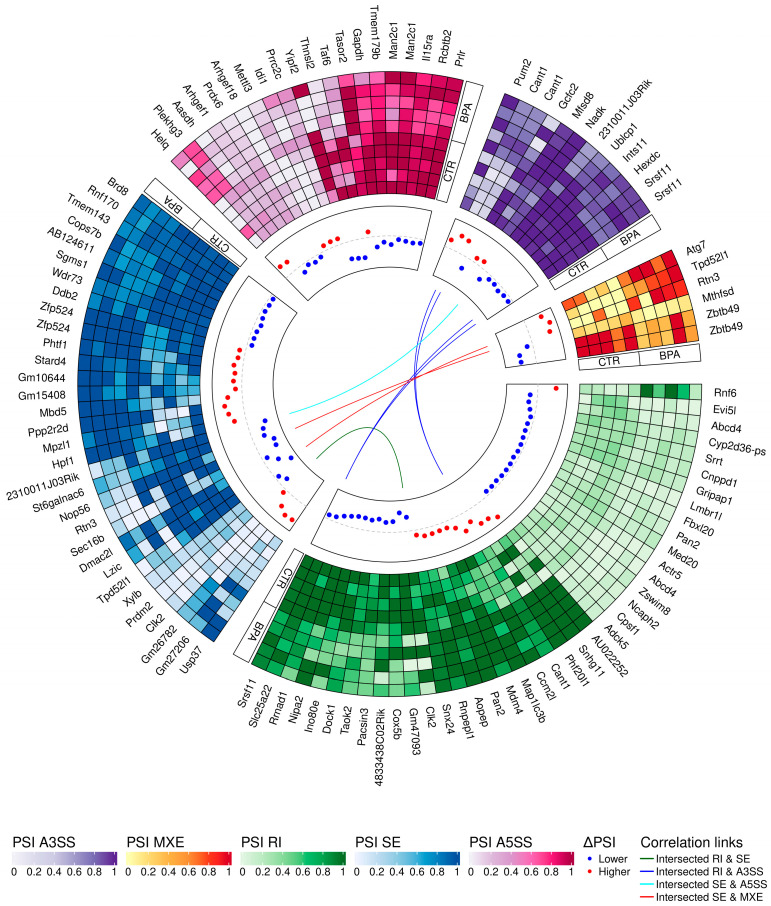
Circular heatmap presents the differentially alternative splicing events (DASs) occurring after BPA treatment. The five-scale color heatmaps (outer track) represent inclusion level values (PSI) in experimental (BPA) and control (CTR) samples. The heatmap blocks present types of alternative splicing events, where purple is alternative 3′ splice site (A3SS), orange is mutually exclusive exons (MXE), green is retained intron (RI), blue is skipped exon (SE), and pink is alternative 5′ splice site (A5SS). The middle track describes the difference in the compared group, measured by inclusion level differences (red—higher inclusion level in BPA, blue—higher inclusion level in CTR). Colors inner links join common genes with more than one DAS classified in different types of alternative splicing events (green line intersects RI and SE, navy RI and A3SS, light blue SE and A5SS, and red links SE and MXE).

**Figure 5:**
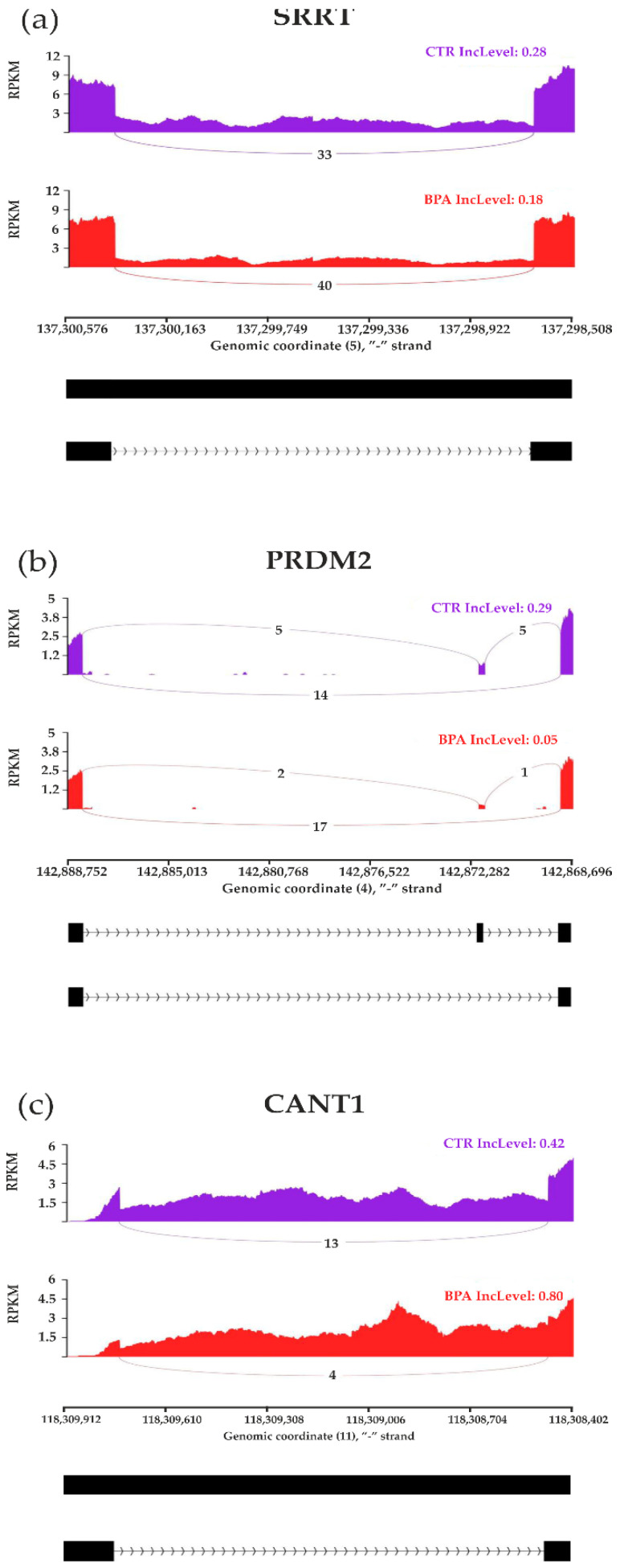
Sashimi plot visualizing statistically significant differentiated alternative splicing events within (**a**) *SRRT*, (**b**) *PRDM2,* and (**c**) *CANT1* genes. Red and purple histograms show detected coverage of RNA-seq reads on the reference genome fragments within control and BPA-treated groups. The average values of reads combining distant genome fragments (black blocks underneath the graphs) are displayed on lines symbolizing spliced regions. Abbreviations: IncLevel—inclusion level, RPKM—reads per kilobase million.

The applied procedure, incorporating rMATS software, allowed the detection of 900 alternative splicing events, including significant 215 DASs resulting from the comparison of BPA vs. CTR samples (Figure 3). Among all detected DASs events, 36 were classified as A5SS, 27 as A3SS, 14 as MXE, 70 as RI, and 68 as SE. The occurrence of alternative splicing significant differences between groups and information on the appearance of multiple events within a single gene are summarized in Figure 4. Calculated inclusion level differences range from −0.71 (SE) to 0.68 (MXE), both within the *TPD52L1*. All disclosed DASs were localized within 187 TARs, of which 90 were identified within protein-coding genes (according to the GCR mouse database), 6 lncRNA-coding regions, and 1 within another genome region. Alternative splicing events were discovered in three DEGs: *DOCK1* (RI, also referred as *MSTRG.23609*), *MSTRG.6242* (three SE events) and *MSTRG.12732* (A5SS), and one DEL: *MSTRG.14959* (SE). Selected events of alternative splicing occurring within *SRRT*, *PRDM2*, and *CANT1* genes are visualized in Figure 5, while all identified cases are summarized in Table S4.

### 3.4. Gene Ontology Networks and Pathway Signaling Analysis of DEGs, DELs and DASs

**Figure 6:**
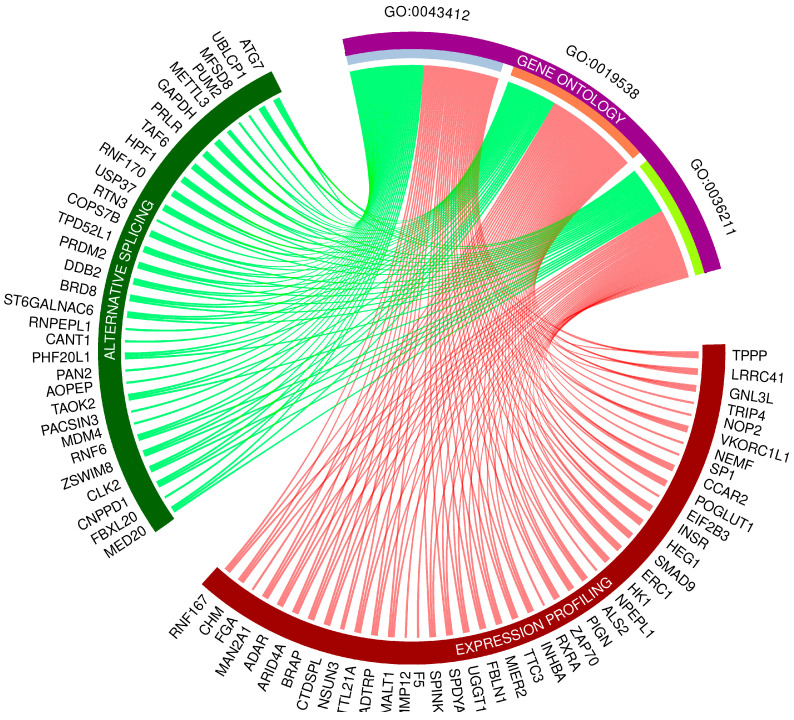
Circos-plot relationship of differentially expressed genes (DEGs) and differentially alternatively spliced genes (DASs) engaged in liver function under BPA influence significantly associated with three selected Gene Ontology (GO)-enriched terms. Color links merge genes with the GO terms (GO:0043412: macromolecule modification; GO:0019538: protein metabolic process; GO:0036211: protein modification process).

Gene Ontology (GO) enrichment analysis reflected the functional annotations of the identified TARs engaged in liver activity after oral BPA administration. The 117/120 unique TARs were assigned to functional GO annotations grouped into eleven BP, two MF, and two CC categories. The GO gene annotation, enriched in BP ontological processes in BPA-affected livers revealed DEGs within terms such as “macromolecule modification” (GO:0043412); “protein metabolic process” (GO:0019538), and “protein modification process” (GO:0036211) (Figure 6). In the MF category, the DEGs were engaged in processes involved in “binding” (GO:0005488) and “peptide hormone binding” (GO:0017046), while the CC category grouping revealed that BPA-modulated mRNAs were involved mainly in “cytoplasm” (GO:0005737) as well as “cytosol” (GO:0005829) functioning. The comprehensive GO enrichment classification is summarized in Table S5.

### 3.5. Validation of the Results

**Figure 7:**
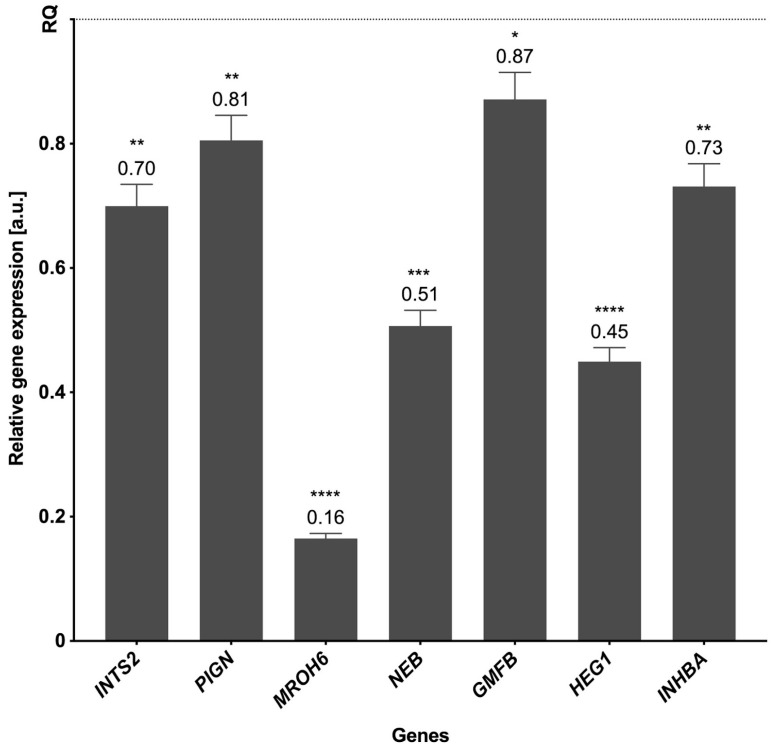
The mRNA expression of selected genes was obtained through real-time PCR. The expression of endogenous control is shown as normalized to a value of 1 (REF), and the expression of validated genes indicates the changes relative to the control. The exact values of the expression are marked above the bars. *p*-values < 0.05 were considered statistically significant, where 0.0332 (*), 0.0021 (**), 0.0002 (***), and 0.0001 (****).

The genes for verification of RNA-seq data were selected following the assessment of their expression values and read’s distribution within liver BPA-treated and CTR samples. Statistical analysis using the Pfaffl method proved the significant changes in the expression levels of seven downregulated genes (*INTS2, PIGN, MROH6, NEB, GMFB, HEG1,* and *INHBA*) compared with the control; real-time PCR analysis confirmed the expression profiles of all validated genes obtained by sequencing (Figure 7).

## 4. Discussion

BPA is regarded as an endocrine-disrupting chemical (EDC) agent. Due to its lipophilicity and occurrence in everyday products, it may be readily absorbed by human beings, leading to anomalous accumulation and malfunction of multiple organs [36]. There is growing evidence that BPA harms liver function, manifested by necrotic changes, inflammatory infiltration, and vascular congestion of hepatocytes [37]. BPA exposure is associated with increased plasma levels of aspartate aminotransferase and alanine aminotransferase, which are considered reliable indicators of hepatocyte damage [38,39]. Recent studies suggest that BPA hepatotoxicity is associated with inducing oxidative stress, mitochondrial damage, and lipid peroxidation resulting from inflammatory responses [39,40]. The development and progression of hepatic tumours have also been linked to BPA accumulation in the body. Modifications of several signalling pathways by BPA accumulation promote cancer cell growth/survival, tissue invasion, and anticancer drug resistance [41]. The results of the current study indicate that oral administration of BPA to living mice results in profound changes in the transcriptomic profiling of key molecular pathways in the liver. The applied multistep bioinformatics screening identified 120 DEGs (including 67 downregulated and 53 upregulated) in the BPA-treated livers, which have been assigned to many biological processes, including “macromolecule modification”, “protein metabolic process”, or “protein modification process”.

BPA administration has been followed by upregulation of the glutathione S-transferase alpha 1 (*GSTA1*) gene encoding glutathione S-transferase alpha 1-1 (GSTA1) protein, which is responsible for the cellular detoxification of endogenous and exogenous compounds in the liver, such as carcinogens, oxidative stress products, and environmental toxins including BPA [42]. Thus, overexpression of *GSTA1* certainly may be caused by ongoing BPA detoxification. Remarkably, excess of GSTA1 is also a very sensitive biomarker for hepatocellular damage [43].

The most striking result emerging from the present study is that several DEGs detected in the liver of mice administered BPA in drinking water for 3 months have been previously associated with severe metabolic liver disorders or malignant tumors. For instance, there was an upregulation of the dedicator of cytokinesis 1 (*DOCK1*) gene in the liver followed BPA intake by mice. The overexpression of *DOCK1* has been observed in various malignancies, including thyroid, bladder, and breast cancers [44,45,46,47]. Most relevant to this study, *DOCK1* upregulation was associated with HCC growth and progression [48]. HCC is one of the five most common cancers in the world [49] that, in contrast to other malignancies, tends to affect only the liver without spreading elsewhere [50]. The development of HCC is multifactorial, including a history of chronic liver disease, hepatitis B virus (HBV), hepatitis C virus (HCV) infection, and alcoholism [51]. 

The transcriptomic analysis made in this study also predicts that exposure to BPA might have carcinogenic potential in the liver. This assumption is strengthened by several DEGs uncovered in the liver of BPA-treated animals, which include overexpression of nuclear factor I B (*NFIB*), NOP2/Sun RNA methyltransferase 3 (*NSUN3*), and serine peptidase inhibitor Kazal-type 1 (*SPINK1*) genes, while transcription of the tubulin polymerization-promoting protein (*TPPP*), inhibin subunit beta A (*INHBA*), heart development protein with EGF-like domains 1 (*HEG1*), and integrator complex subunit 2 (*INTS2*) genes were downregulated. It is known that *NFIB* is an oncogene, and, according to recent reports, its upregulation has been linked with the growth and progression of several types of cancer [51], including HCC [52]. A concerning overexpression is that of the *NSUN3* gene, as it has been involved in the promotion of cancer cell invasion and metastasis by favoring the synthesis of proteins participating in the mitochondrial respiratory chain [53]. Excessive *NSUN3* transcription has been positively correlated with poor prognosis in patients with HCC [53]. Likewise, overexpression of *SPINK1* (also known as tumor-associated trypsin inhibitor, *TATI*) has been linked to the expansion of many malignant tumors, including the HCC, as it favors cancer cells proliferation, metastasis, and anticancer drug resistance [54,55,56,57]. Also, the protein encoded by the *TPPP* gene plays a role in cancerogenesis [58], as it controls normal cell proliferation by modulating microtubule dynamics [59]. Therefore, BPA-induced downregulation of *TPPP* gene transcription may contribute to abnormal hepatic cell proliferation and poor prognosis of patients with HCC, as previously demonstrated [60]. The downregulated INHBA encodes inhibin protein belonging to the TGFβ superfamily, which controls cell proliferation, differentiation, apoptosis, and inflammation in many cell types and organs including the liver, and recently, it has been found that INHBA is strongly engaged in hepatocarcinogenesis in rats [61,62,63]. The under-expressed HEG1 encodes protein participating in the cancer metastasis process [64], while the protein encoded by the downregulated INTS2 belongs to a key regulator of RNA polymerase II-mediated transcription integrator complex. The functional impairment of this complex has been revealed in several types of tumors [65,66].

It is worth mentioning that we also examined differential alternative splicing (DAS) gene events in the livers of healthy controls and BPA-treated mice. Our findings show that some identified DAS gene events, including helicase POLQ-like (*HELQ*), StAR-related lipid transfer domain containing 4 (*STARD4*), cytochrome C oxidase subunit 5B (*COX5B*), serrate, RNA effector molecule (*SRRT*), positive regulatory/Su(var)3-9, enhancer-of-zeste and trithorax domain 2 (*PRDM2*), and calcium-activated nucleotidase 1 (*CANT1*) were previously associated with cancerogenesis. HELQ is critical for DNA repair, thus, it is regarded as an important genome caretaker and has been identified as a potential target for anticancer therapies. Moreover, emerging evidence reveals that *HELQ* alterations leads to abnormalities in its protective action, which are often found in malignancies, such as squamous cell carcinoma, gastric cancer, and HCC [67]. *STARD4* gene transcription is upregulated in patients suffering from HCC, in which its levels correlate with poor prognosis. Presumably, *STARD4* transcriptional variations in HCC significantly affect cholesterol intracellular metabolism and transport [68]. The *COX5B* gene is highly expressed in HCC, thus, confirming its putative role as a growth-promoting agent in this type of cancer. Upregulation of *COX5B* gene transcripts in hepatoma cells is also associated with an unfavorable postoperative prognosis of HCC, concurring with increased proliferation and migration of this tumor type [69]. The *SRRT* gene (also known as an arsenite-resistance protein 2 gene; *ARS2*) is critical in mammalian cell proliferation. Knockdown of this gene slows all stages of the cell cycle, whereas its overexpression promotes HCC and has a prognostic value in this type of cancer [70]. Another alternatively spliced gene revealed in our study, *PRDM2,* is a tumor suppressor gene, downregulated in various cancers. *PRDM2* belongs to HCC-associated genes and it has been revealed that its hypermethylation significantly increases the risk of HCC [71]. *CANT1* is also known to be overexpressed in several malignancies and its alternative splicing transcript variants in tumors are considered important indicators of cancer progression [72]. Knockdown of this gene causes suppression of cell proliferation, migration, and invasion, thus, it has been proposed as a potential therapeutic target for HCC [73]. 

Furthermore, some uncovered DAS gene events were localized within transcripts involved in autophagy, such as the microtubule-associated protein 1 light chain 3 beta (*MAP1LC3B*; a gene known to be an autophagy marker strongly expressed in HCC cells) [74] and the autophagy-related 7 gene (*ATG7*; encoding a protein working in the autophagy pathway as a ubiquitin-activating enzyme) [75]. Given that BPA intake has been positively linked to oxidative stress and ROS formation [11] and the latter processes initiate autophagy designed to remove unnecessary or damaged cellular components [76], alternative splicing of genes involved in autophagy may result in the accumulation of dysfunctional proteins, leading to organ failure and cancer development [76].

Several studies have indicated the linkage between BPA tissue contamination and the incidence of non-alcoholic fatty liver disease (NAFLD) [20,77]. NAFLD is the major cause of chronic liver disease among children and adults and the most common cause of liver transplantation [78]. The NAFLD pathological spectrum goes from simple steatosis to steatohepatitis, which may progress to hepatic cirrhosis and HCC [79]. NAFLD is strongly associated with obesity, insulin resistance, and type 2 diabetes mellitus [80]. Excessive caloric intake leads to increased serum levels of free fatty acids (FFA) and adipocyte resistance to insulin. Insulin resistance contributes to NAFLD by damaging the insulin receptor signaling, causing the defective inhibition of FFA release from fat cells. Therefore, insulin resistance and excess FFA are thought to be a vicious circle in the development of NAFLD [81,82]. The RNA-seq analysis performed in this study shows that oral exposure to BPA identified several DEGs in the liver contributing to NAFLD development. These include the downregulation of the insulin receptor (*INSR*), retinoid X receptor alpha (*RXRA*),pantothenate kinase 1 (*PANK1*), and maestro heat-like repeat family member 6 (*MROH6*). INSR is a receptor that upon binding to insulin regulates the management of proteins, lipids, and carbohydrates, including glucose absorption and cellular uptake [81]. Deficient *INSR* gene processing may impair insulin biological responses, ultimately causing insulin resistance and glucose intolerance [83,84]. On the other hand, both *RXRA* and *PANK1* are involved in signaling pathways controlling the metabolism of lipids by hepatocytes [85,86], while downregulation may contribute to serious liver disorders, such as NAFLD. Moreover, *PANK1* is a negative regulator of the Wnt/β-catenin signaling cascade involved in the pathogenesis of HCC. *PANK1* has been pointed out as a potential therapeutic target for HCC, given that downregulation of this gene transcription promotes the growth and invasiveness of HCC cells [87]. The alterations of the *MROH6* have been recently revealed in pre-teenage children suffering from NAFLD [88].

## 5. Conclusions

To the best of our knowledge, this is the first study addressing the impact of oral intake of BPA on gene transcription changes in the liver to predict the molecular mechanisms underlying hepatic toxicity and potential carcinogenicity of this widespread environmental chemical contaminant. Data from our comprehensive transcriptomic analysis together with diverse sets of results from the literature confirm that exposure to BPA exhibits a carcinogenic potential, as well as contributing to the development of other severe metabolic liver diseases, including NAFLD. The authors are aware that exposure to BPA starting at a younger age of mice would be more reflective of real-world exposure scenarios and that post-weaning animals are much more sensitive to the effects of toxic compounds, however, the aim of the study was to evaluate the influence of BPA on the adult, no-longer-developing organism. Moreover, it must be emphasized that young cells have greater regenerative capabilities so, in order to better understand the hepatotoxic effects of this xenoestrogen after a long-time exposure, the authors decided to study post-maturation organisms. Of course transcriptomics prediction of HCC and NAFLD from BPA exposure may not be of clinical relevance, however, in our opinion, it should constitute a legitimate ground for further reducing its usage, especially in food containers.

## Figures and Tables

**Table 1:** Overview of the sequencing and mapping results for the ten RNA-seq libraries. CTR 1–5 refers to the biological replicates of the control liver; BPA 1–5 refers to the mice liver after BPA administration; unique reads refer to the reads mapped to only one location of the mouse genome; multi-mapped reads refer to reads aligned to more than one locus on the reference genome; reads mapped with too many loci refer to the reads mapped more than 20 to multiple loci on the reference genome.

Sample	CTR1	CTR2	CTR3	CTR4	CTR5	BPA1	BPA2	BPA3	BPA4	BPA5
**Raw reads**	63,701,054	62,907,658	63,490,610	61,783,238	65,647,032	61,466,386	63,656,704	60,608,086	64,195,852	65,720,614
**Processed reads**	53,607,300	52,885,042	54,905,736	52,410,248	56,702,958	51,304,764	55,754,414	52,413,416	54,694,930	56,629,844
**All mapped reads**	48,317,860	44,886,888	50,268,208	48,843,756	51,240,802	48,998,204	51,833,736	49,518,666	50,821,082	51,468,034
**Uniquely mapped**	44,535,610	41,312,746	46,211,692	44,860,506	47,096,330	45,390,806	47,870,430	46,095,184	46,905,746	47,462,692
**Multi-mapped reads**	3,767,304	3,554,566	4,035,672	3,960,676	4,120,960	3,593,654	3,943,166	3,406,710	3,898,412	3,992,284
**Mapped to too many loci**	14,946	19,576	20,844	22,574	23,512	13,744	20,140	16,772	16,924	13,058
**% of CDS mapped bases**	67.74%	68.16%	68.71%	67.99%	68.70%	70.27%	68.98%	68.11%	69.05%	69.02%
**% of UTR mapped bases**	27.01%	26.96%	26.37%	26.92%	26.59%	26.44%	27.35%	28.11%	27.42%	26.86%
**% of Intron mapped bases**	3.37%	3.01%	3.05%	3.31%	2.96%	1.86%	2.05%	2.23%	1.99%	2.37%
**% of Intergenic mapped bases**	1.88%	1.87%	1.86%	1.79%	1.74%	1.43%	1.63%	1.55%	1.54%	1.74%

## Data Availability

The RNA-seq data have been submitted (https://www.ebi.ac.uk/ena, accessed date: 14 August 2023) to the European Nucleotide Archive under accession no. PRJEB (https://www.ebi.ac.uk/ena/browser/view/PRJEB65086, accessed date: 1 October 2023).

## Supplementary Materials

The following supporting information can be downloaded at: https://www.mdpi.com/article/10.3390/cancers15205014/s1, Table S1: The list of primers applied during real-time PCR procedure. Table S2: Detailed results of DEGs, DELs, and other DE-RNAs expression patterns induced by BPA treatment in mouse liver; Table S3: Results of DEGs–DELs and DEGs–other DE-RNAs trans-interactions; Table S4: Detailed results of BPA-modulated DASs analysis in mouse liver; Table S5: Results of GO, Reactome, and TRANSFAC functional analysis of DEGs and genes incorporating DASs induced by BPA treatment in mouse liver.
