# Supplementary material for: The Carcinogenic Potential of Bisphenol A in the Liver Based on Transcriptomic Studies
Source: Cancers (Basel). 2023 Oct 17;15(20):5014. doi: 10.3390/cancers15205014 (PMC10605469; doi:10.3390/cancers15205014)
Supplement: Supplementary file 1 [file cancers-15-05014-s001.zip › Table S1.pdf]

Table S1. The list of primers used for Real-time PCR.

Name	Forward sequence: (5' to 3')	Reverse sequence: (5' to 3')
INTS2	CCTCTTGCCCTGCCTGGTGA	GCTCTCTCCACTGCCTCCTCC
PIGN	TGATCCAAGCCTGTCCCTGGA	TGAGAAACGGCCAGCCTGCA
MROH6	GCCACTCGCGCTCTTGGAGA	AGGCCTCCACTGTGCAGCTG
NEB	TGGGATCCAGCACGCCAAGG	CCCTCCTTGGCGGCGTTGAT
GMFB	CCTGGTGGTGCTGGATGAGGA	TGGTTAGCTCGGCAGTCTGGAC
HEG1	CACCGTCACAACCCCAGCCA	TCACACGTGGCTCCCTTGGC
INHBA	CAAGGCGGCGCTTCTCAACG	GCCACACTTCTGCACGCTCC
